# Simulating a flexible water model as rigid: Best practices and lessons learned

**DOI:** 10.1063/5.0143836

**Published:** 2023-04-03

**Authors:** Raymond Weldon, Feng Wang

**Affiliations:** Department of Chemistry and Biochemistry, University of Arkansas, Fayetteville, Arkansas 72701, USA

## Abstract

Two ways to create rigid versions of flexible models are explored. The rigid model can assume the Model’s Geometry (MG) as if the molecule is not interacting with any other molecules or the ensemble averaged geometry (EG) under a particular thermodynamic condition. Although the MG model is more straightforward to create, it leads to relatively poor performance. The EG model behaves similarly to the corresponding flexible model (the FL model) and, in some cases, agrees even better with experiments. While the difference between the EG and the FL models is mostly a result of flexibility, the MG and EG models have different dipole moments as a result of an effective induction in the condensed phase. For the three water models studied, the property that shows the most difference is the temperature dependence of density. The MG version of the water model by adaptive force matching for ice and liquid does not possess a temperature of maximum density, which is attributed to a downshift of the putative liquid–liquid phase transition line, leading to the hypothesized second critical point of liquid water to manifest at negative pressure. A new three-phase coexistence method for determining the melting temperature of ice is also presented.

## I. INTRODUCTION

Many water models ignore the geometrical flexibility of the molecule and still provide a good description of various properties of this challenging material.^1–3^ This is not surprising as a typical O–H bond in water is expected to vibrate in a range of 0.03 Å at room temperature in a Newtonian mechanics-based simulation. In fact, the classical Hooke’s picture of intramolecular vibrations is incorrect considering the quantum nature of these vibrations, causing flexible models to be worse for certain properties,^4^ such as the heat capacity. At the same time, there are many flexible water models that are successful by showing good agreement with experiments. It would be desirable to simulate these flexible water models as rigid, as such a practice will greatly improve computational efficiency. Modeling the intramolecular OH vibrations requires small integration time steps, such as 0.5 fs, whereas a 2 fs time step can be used with rigid models. Thus, simply removing intramolecular vibrations would lead to a factor of 4 saving in central processing unit (CPU) time, making microsecond trajectories much more accessible. Microsecond trajectories are important when properties at low temperature are to be investigated.

A systematic study of the effect of eliminating the flexibility of a semi-rigid model is lacking in the literature. It is not entirely clear which physical properties are most affected by explicitly modeling the vibrations of the OH bond and HOH angle in water. In force field development, constraining stiff vibrations as rigid is a routine practice,^5–7^ especially for small molecules. Investigation of the effects of molecular flexibility would guide future design of such force fields.

In this study, we explore two different practices for generating rigid versions of a flexible model. As will be discussed later, similar considerations also apply when a flexible version of a rigid model is created. Although we will restrict ourselves to water models the general principle should apply to other semi-rigid small molecules. We showed that the most straight-forward model geometry based approach for generating rigid models would lead to systematic errors, whereas simply using the mean geometry under the desired thermodynamic condition will lead to a large improvement in measured properties. Some properties, such as temperature dependence of density, are found to be quite sensitive to small differences in the rigid body geometry.

To confirm the generality of our conclusion, we created rigid versions of three different flexible water models, BLYPSP-4F,^8^ Water model by Adaptive force matching for Ice and Liquid (WAIL),^9^ and q-TIP4P/F.^10^ The first two models were developed with the adaptive force matching (AFM) method^11^ by fitting to coupled cluster quality reference forces.^12^ The q-TIP4P/F model was developed by fitting to selected experimental properties in a path-integral simulation. In this work, no path-integral is used when the properties of different versions of the q-TIP4P/F model are studied as the objective is only to see the effect of rigidity. In addition, we will present a new way to determine the melting temperature of a water model more reliably.

The study will be presented in five sections. After the Introduction, the methods for generating rigid versions of a flexible model are presented in Sec. II. The new procedure to determine the melting temperature of ice along with other simulation details is reported in Sec. III, followed by results and discussions in Sec. IV and the summary and conclusions in Sec. V.

## II. METHODS FOR CREATING A RIGID VERSION OF A FLEXIBLE MODEL

The relative merits and shortcomings of flexible and rigid models have been studied before,^4,13^ including analysis on the effects of flexibility for properties such as viscosity, phase behavior near criticality, and gas phase cluster formation kinetics.^14–16^ In some of the studies, the flexible and rigid models do not share the same intermolecular terms, thus confounding the effect of flexibility and the differences in intermolecular potentials. Flexible versions of rigid models have been created, and the variation of molecular dipole moments between rigid and flexible versions of the same model has been pointed out.^17^ However, none of the previous studies, to the best of our knowledge, have noted any substantial effects of flexibility beyond what could be contributed to a small enhanced dipole of the flexible model. Our work studied a wide range of properties and revealed that certain properties depend strongly on the protocol for creating a rigid version of a flexible model.

The most straightforward way to create a rigid model is to use the Model Geometry (MG) of the force field. In this case, the geometry of an isolated monomer will be maintained with a restraint algorithm^18,19^ or rigid body integrator.^20^ The model geometries for each of the three force fields tested are summarized in Table I. The rigid models created with these geometries will be referred to as the MG models.

**TABLE I.** Geometries and dipole moments of the rigid water models. All the models are four site models with the OM distance listed in this table. Further details of the models can be found in the supplementary material. The MG models use the geometry of the force field. The EG models use the ensemble averaged geometry measured from NPT simulations at 298 K and 1 bar.

	Model	Bond length (Å)	Bond angle (deg)	Dipole (D)	OM distance (Å)
MG	BLYPSP-4F	0.951	106.678	2.175	0.227
	WAIL	0.9496	106.890	2.238	0.226
	q-TIP4P/F	0.9419	107.400	2.194	0.147
EG	BLYPSP-4F	0.9697	105.260	2.254	0.235
	WAIL	0.9707	105.494	2.325	0.235
	q-TIP4P/F	0.9635	104.670	2.316	0.155

As a result of intermolecular interactions in the condensed phase, the average geometry of a flexible water model in both the liquid and solid states will deviate from the model geometry.^17,21^ All flexible water models are effectively polarizable. The bending of the HOH angle will increase the dipole of water leading to an in-plane polarizability,^21^

$$\alpha =\frac{q_{H}^{2}r_{OH}^{2}\left(1-2a\right)}{k_{\theta }},$$ (1)

where *q*_H_ is the charge on the H atom, *r*_OH_ is the OH bond length, and *a* is the parameter that defines the M site based on OH bond vectors,

$${\vec{r}}_{M}={\vec{r}}_{\text{O}}+a\left({\vec{r}}_{\text{O}H_{1}}+{\vec{r}}_{\text{O}H_{2}}\right),$$ (2)

and *k*_*θ*_ is the vibrational force constant for the HOH bend.

Due to the effective polarizability, an average water molecule in the condensed phase has a smaller HOH angle than that of the model; similarly, the average bond length is also longer.^22^ The response of the molecular shape to the condensed phase environment leads to a greater average dipole moment than that of the model itself. Intuitively, using such average geometries will produce rigid models that give ensemble properties in better agreement with their flexible counterparts.

It is worth noting that the average geometries depend on simulation conditions, such as temperature and pressure. It has been proposed that the average water geometry can be used as a proxy to measure pressure in a nanodroplet.^23^ In addition, the average geometry of water in ice will be different from that in the liquid phase,^24^ although the ensemble dependence of geometry is expected to be weak. The use of an average geometry will be convenient only if substantial improvement in water properties can be obtained when the geometry of a nearby reference thermodynamic condition is used.

In this work, we choose liquid water at 1 bar and 298 K as the reference thermodynamic condition. A detailed procedure for measuring the ensemble geometry (EG) will be provided in Sec. III. The average geometry of the reference ensemble will be used for simulations of water in a range of thermodynamic conditions, including ice and liquid. The average ensemble geometries are also summarized in Table I. The corresponding rigid models based on this geometry will be referred to as EG models, which stand for ensemble geometry.

It should be noted that the rigid version of the models constrains the bond length and angles but otherwise uses identical parameters as the flexible model, including the locations of the M site. For convenience, all model parameters and the definition of the M site are supplied in the supplementary material.

## III. SIMULATION DETAILS AND METHOD FOR MEASURING THE MELTING TEMPERATURE OF ICE

For measuring the average geometry of the EG model, a box containing 1728 water molecules was simulated for 3 ns at 298 K and 1 bar. The geometry was extracted with MDTraj^25^ 1.9.7 based on intramolecular OH and HH distances. The long range Coulombic interactions are described with particle mesh Ewald.^26^ Van der Waals interactions are cutoff at 1.0 nm. The long range corrections to energy and pressure due to the truncation of van der Waals are applied for the BLYPSP-4F and q-TIP4P/F models. While most AFM models were developed by fitting dispersion separately to Symmetry Adapted Perturbation Theory (SAPT)^27^ or Grimme’s empirical formula,^28^ WAIL is one of the models where the dispersion term was fit along with other intermolecular terms without a separate step. Due to the use of a small quantum mechanics (QM) cluster and the coupling of the 1/*r*^6^ term with repulsion, the C_6_ determined with such a procedure is less reliable than that from a separate SAPT fit. Consequently, the WAIL potential is better used without long range correction to dispersion.

Since a rigid model simulation would not be able to capture the small geometry difference between water in ice and liquid phases, we are particularly interested in studying the effect of using a rigid model for simulations of ice–water equilibrium. Supercooled water and ice are two systems where the need for long trajectories is most compelling and can benefit the greatest from rigid versions of the models.

One of the most straightforward methods for determination of the melting temperature, *T*_*M*_, is the direct coexistence method.^29^ Our simulations have encountered some instability with the coexistence method as the *T*_*M*_ depends on choices and relaxation parameters of the barostat. A typical barostat will scale the coordinates of all atoms. This is true even for anisotropic barostats. Although such barostats use different scaling factors for different lattice directions, in each direction, both the ice and water coordinates are scaled at the same rate, ignoring the differences in the compressibilities of the solid and liquid phases. The substantial change in the density associated with the phase change of ice poses a particularly large challenge. Rather than localized expansion at the growth interface, a typical barostat will scale uniformly in the direction normal to the growth surface, which is not entirely physical.

In this work, we avoid the use of both barostats and thermostats by simulating an ice–water mixture in the microcanonical ensemble. The method will be referred to as the NVE three-phase coexistence method. The idea is to prepare liquid–ice–liquid (LIL) slabs at two or more temperatures near the melting temperature *T*_*M*_ of the model. The two liquid regions are separated by a vapor phase to allow expansion and contraction during phase changes. At least two simulations starting from different temperatures are needed to judge convergence. We will refer to such temperatures as the target temperatures and label the two target temperatures as *T*_*H*_ and *T*_*L*_. The target temperature is the initial temperature of the NVE simulation. If the initial temperature is above *T*_*M*_, the slab will be cooled down as ice melts. If the initial temperature is below *T*_*M*_, ice will grow and the exothermic freezing process will warm up the slab. Expansion and contraction during phase transitions are accommodated with the vapor region.

The liquid is made sufficiently thick so the liquid–vapor interface would not affect the ice–water interface. The lateral dimensions are determined by the density of ice at target temperatures near *T*_*M*_. The lateral pressure of such a slab should not be zero due to the presence of interfaces and associated surface tension. We ensure that ice in the LIL slab has the *a*, *b* lattice vectors being that of the bulk ice at 0 bar. The normal pressure on the slab is the equilibrium vapor pressure at the triple point of water, which is for all practical purposes, 0 bar as far as our simulation is concerned. The *T*_*M*_ of ice only has a weak dependence on pressure. At 0 bar, the *T*_*M*_ is only 0.01 K higher for real water. Thus, we believe measuring the *T*_*M*_ at 0 bar is sufficient.

In our study, six LIL slabs were built from six ice initial conformations and run at *T_H_* and *T_L_* to determine *T*_*M*_. The six slabs were created following the electrostatic switching method of Lindberg and Wang^30^ to sample different proton orientations. Ice is proton disordered with a long relaxation time for proton rearrangements. Using the electrostatic switching method to create initial configurations allows such proton orientational degrees of freedom to be properly averaged.

Each proton disordered ice box containing 300 water molecules is stacked four times in the z-dimension. The stacked ice box without any liquid or vacuum will be equilibrated at the target temperature and 0 bar for 1 ns with temperature and pressure controlled using the Nosé–Hoover thermostat and Parrinello–Rahman barostat. The relaxation times for the thermostat and barostat are 2 and 5 ps, respectively. The average box dimensions were determined by averaging over the last 0.9 ns of the simulations. The box size determination will be performed at every target temperature for each of the six proton disordered configurations for each water model.

A vacuum of 1 nm is added to the equilibrated ice box at the average box dimension. The 600 ice molecules at the center of the slab are frozen, and the 300 water molecules on each side are allowed to melt at T_melt_ for 200 ps in an NVT simulation. It should be noted that the T_melt_ is the temperature used to melt the ends to create the LIL slab and it is not the *T*_*M*_ to be measured. The T_melt_ for each model is summarized in Table II. After the ends are melted, the slab will be cooled for 200 ps to the target temperatures for the *T*_*M*_ study. In the first 100 ps of the cooling, the 600 ice molecules remain frozen; in the second 100 ps, all 1200 water molecules will be equilibrated at the target temperature. The melting and cooling steps will be performed with a stochastic rescaling thermostat^31^ with a 0.5 fs relaxation time.

**TABLE II.** Temperatures used for the NVE three phase coexistence method. T_melt_ is the temperature used to melt the ends of the ice slab. *T_H_* and *T_L_* are target temperatures for starting the NVE simulations.

	Model	T_melt_ (K)	T_L_ (K)	T_H_ (K)
FL	BLYPSP-4F	300	245	265
	WAIL	320	260	280
	q-TIP4P/F	300	245	265
MG	BLYPSP-4F	300	230	250
	WAIL	320	240	260
	q-TIP4P/F	300	225	245
EG	BLYPSP-4F	320	245	265
	WAIL	340	260	280
	q-TIP4P/F	300	245	265

Six ice boxes of WAIL water created to sample different proton orientations are provided in the supplementary material, along with the corresponding LIL slabs, to facilitate subsequent studies using the NVE three-phase coexistence method.

Table II also summarizes the final *T*_*H*_ and *T*_*L*_ used for each model. *T*_*H*_ and *T*_*L*_ should be sufficiently close to the *T*_*M*_ to prevent the LIL slab from completely melting or freezing over. Such incidences can easily be detected by visual inspection of the slab or the temperature trace. The LIL temperature from the two simulations will not agree even after a long period of time if one of the configurations no longer has solid–liquid coexistence. We pick *T*_*H*_ to be above the *T*_*M*_ and the *T*_*L*_ to be below *T*_*M*_, although this is not strictly required. It is convenient for us since our previous work on these models already provided us with a reasonable guess of the *T*_*M*_. For water models with unknown *T*_*M*_, few explorative NVE three-phase coexistence simulations will allow *T*_*M*_ to be approximated quickly.

**FIG. 1.**
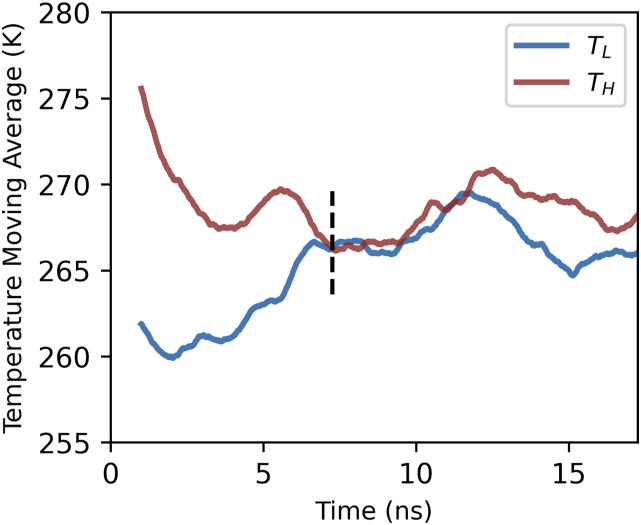
Example trajectory of an NVE three phase consistence simulation. Solid lines are the 1 ns rolling average of the temperature. *T_L_* and *T_H_* are the initial temperatures. The vertical dashed line marks the beginning of the region considered to have reached equilibrium. The average temperature of the 10 ns trajectory after equilibrium is the estimated *T*_*M*_. We note that *T*_*M*_ reported in this work is averaged over six different proton disordered initial conformations.

To judge equilibrium, a 1 ns rolling average of the temperature will be monitored and equilibrium is considered to be reached once the rolling averages from the two initial conditions are within 0.1 K of each other. Once equilibrium is reached, the temperature is averaged for 10 ns. The average temperature in this 10 ns window is *T*_*M*_. Figure 1 shows an example of such a temperature trace for the flexible WAIL potential. We note the temperature fluctuations observed in Fig. 1 after equilibrium has been reached are expected. Water spontaneously transforms between ice and liquid even at equilibrium; such transformations will lead to short term elevation and depression of the instantaneous temperature in an NVE ensemble.

We also report the total energy as a function of time for 20 ns in the supplementary material. The NVE implementation in Gromacs has no problem conserving total energy with a 0.5 fs time step size. We note that a slow drift in total energy can be tolerated as long as there is still solid–liquid coexistence as the potential energy change associated with the phase transition will absorb the energy loss or gain from an imprecise integrator.

To provide proper validation of the NVE three-phase coexistence method, the *T*_*M*_ of the TIP4P/Ice^32^ model was measured. With six proton disordered initial conformations and averaging over the 12 NVE trajectories, the *T*_*M*_ of TIP4P/Ice was determined to be 274 ± 1 K, which is in good agreement with prior estimates ranging from 268 to 272 K.^32–34^ Of the previous estimates, two values based on free energy methods gave *T*_*M*_ from 270 to 272 K,^32,34^ whereas two estimates based on the two phase direct coexistence method gave *T*_*M*_ in the range from 268 to 270 K.^33,35^ The typical direct coexistence method maintains the lateral pressure of the ice–liquid slab close to zero. As the ice–liquid interface possesses a surface tension, the ice region will be under a positive lateral stress in order to balance the contraction at the interface. The compression of ice could slightly depress the *T*_*M*_ of the two-phase coexistence method. The density of ice in the NVE-three-phase coexistence method is not affected by the surface tension, which is consistent with a T_*M*_ in closer agreement to free energy based estimates.

In addition to *T*_*M*_, the radial distribution functions (RDFs), density as a function of temperature, surface tension (*γ*), dielectric constant (*ε*), heat capacity (*Cv*), heat of vaporization (*ΔH*_*vap*_), and diffusion constant (*D*) are measured for each model.

Unless otherwise noted, all simulations were performed using Gromacs 2019.6 with a constant number of particles at 1 bar and 298 K. The temperature and pressure were controlled with Nosé–Hoover thermostat and Parrinello–Rahman barostat, with 2 and 5 ps relaxation times, respectively. Long range corrections to van der Waals were used for the BLYPSP-4F and q-TIP4P/4F models.

All flexible models were simulated with a 0.5 fs time step, and all rigid models were simulated with a 2 fs time step except for the NVE steps of the *T*_*M*_ study. NVE simulations with a 2 fs time step are challenging due to energy drifts. Thus, a 0.5 fs time step was used for all NVE simulations regardless of model flexibility. A 1 fs time step might be sufficient for rigid body NVE simulations, but we have not fully tested this.

The linear constraint solver (LINCS) algorithm^18^ is used to constrain the rigid water molecules. A 12th order expansion of the constraint coupling matrix is used with six LINCS iterations.

**FIG. 2.**
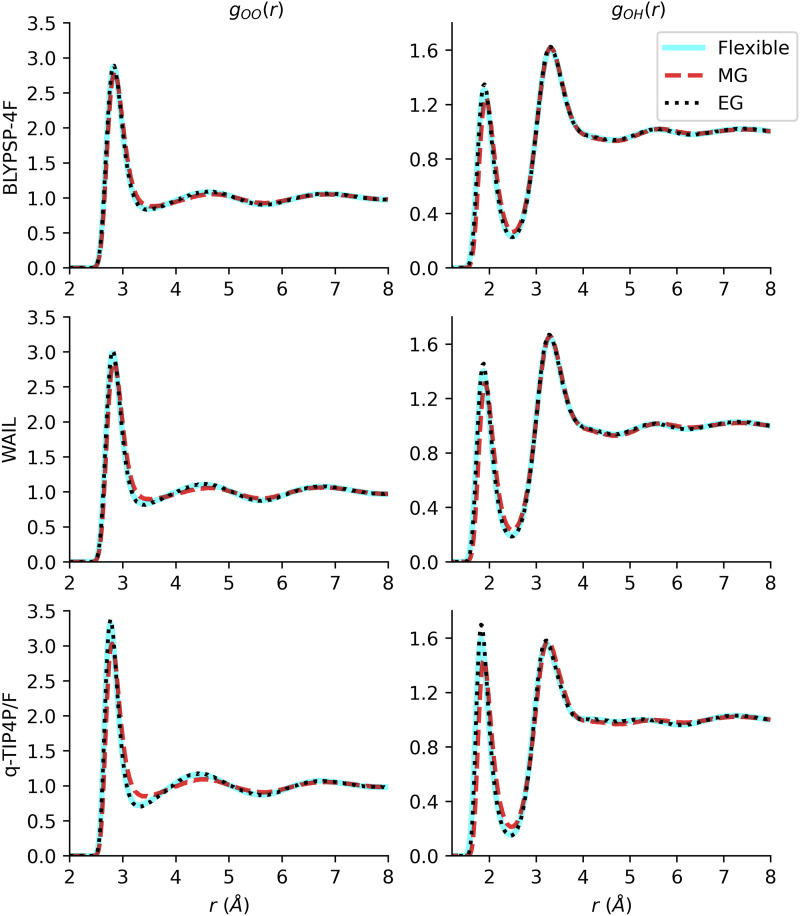
Radial distribution functions of the BLYPSP-4F (top), WAIL (middle), and q-TIP4P/F (bottom) models. Ignoring intramolecular vibrations has little effect on the intermolecular RDFs.

The density as a function of temperature is measured using a 343 water box. Data within a 20 K range around the density maximum are used to fit the temperature of maximum density (TMD) of each model. For each temperature shown in Fig. 2, at least 100 ns of NPT simulations were performed to obtain the density.

The *ε* was computed from the variance of the box dipole, *M*, using the formula from the fluctuation and dissipation theorem:^36^

$$\varepsilon_{o}=1+\frac{4\pi }{3Vk_{B}T}\left(\left\langle M^{2}\right\rangle -{\left\langle M\right\rangle }^{2}\right),$$ (3)

where *V* is the volume of the box and the temperature T is 298 K. Since none of the force fields studied in this work has explicit polarizability to model electronic polarization, Eq. (3) only includes the orientational contribution to the *ε*. Thus, it would not be appropriate to compare such an *ε*_*o*_ with the experimental *ε* of water, which also includes the high frequency component of the dielectric due to electronic response. We report also in Table III, the *ε*, which is 1.78 × *ε*_*o*_, to account for the high frequency component of the water dielectric constant.^37^

**TABLE III.** Properties of the models studied along with the corresponding experimental values. The dielectric constant of the model, *ε*_*o*_, is multiplied by the high frequency component of 1.78 and reported as *ε*. Since the experimental value includes the high frequency component of the dielectric constant, it is listed in the column for *ε*. Error bars for *T*_*M*_ are the standard error of the mean.

		*T*_*M*_ (K)	Δ*H*_*vap*_ (kJ/mol)	*D* (10^−5^ cm^2^/s)	ϒ (mN/m)	*ε*_*o*_	*ε*	TMD (K)	*Cv* (J/mol K)
BLYPSP-4F	FL	252 ± 1	48.4	2.47	67.0	45.1	80.3	267.6	103.1
	MG	237 ± 1	46.9	3.03	63.6	40.4	71.9	239.1	75.5
	EG	255 ± 1	49.6	2.20	68.2	44.3	78.8	271.0	78.0
WAIL	FL	267 ± 1	53.4	1.53	76.9	48.6	86.5	273.0	103.6
	MG	245 ± 1	51.4	2.09	73.2	43.3	77.1	n/a	75.5
	EG	272 ± 2	54.7	1.31	78.2	48.1	85.6	279.0	79.5
q-TIP4P/F	FL	257 ± 1	46.3	1.99	67.4	60.5	107.8	281.0	115.0
	MG	237 ± 1	43.3	3.30	60.4	50.5	89.9	253.1	81.5
	EG	261 ± 2	47.4	1.68	69.4	55.0	98.0	286.5	91.8
Expt.^40,41^		273.15	43.98	2.30	72.06		78.36	277	74.6

*Cv* was estimated with the finite difference method using two different NVE simulations using configurations sampled from an NVT trajectory. Due to the fluctuations in the NVT simulation, from any instantaneous configuration, the average temperature of an NVE simulation starting from such a configuration will not be 298 K but will remain fairly close. The *Cv* was computed with the equation

$$Cv=\frac{\Delta E}{\Delta T},$$ (4)

where *ΔT* is the difference in the mean temperature of the two NVE simulations. The two NVE simulations were performed for 4 ns each using 1728 water boxes.

The *γ* was computed from a 1728 water liquid slab using an orthorhombic box with a 6 nm vacuum between the two interfaces. The Coulombic interaction was modeled with standard 3D particle mesh Ewald, and a long 1.75 nm van der Waals cutoff was used for BLYPSP-4F and q-TIP4P/F models.^38^

The *ΔH*_*vap*_ was computed with the following formula:

$$\Delta H_{\mathrm{vap}}=\left\langle V_{\mathrm{gas}}\right\rangle -\left\langle V_{\mathrm{liq}}\right\rangle -\Delta E_{\mathrm{self}}+RT,$$ (5)

where *V*_*liq*_ is the potential energy of the liquid phase and *V*_*gas*_ is the intramolecular configuration energy in the gas phase. The angle bracket indicates ensemble average. Since the models used a dipole moment larger than the gas phase dipole to implicitly include polarization effects in the condensed phase, the polarization self-energy correction,^3^ *ΔE*_*self*_, is used in Eq. (5). *ΔE*_*self*_ is computed with

$$\Delta E_{\mathrm{self}}=\frac{{\left(\mu_{m}-\mu_{g}\right)}^{2}}{2\alpha },$$ (6)

where *μ*_*m*_ is the model dipole moment, *μ*_*g*_ is the experimental dipole moment of water in the gas phase, and the polarizability, *α*, is 1.44 Å^3^.^39^ The calculated *ΔE*_*self*_ for each model is provided in the supplementary material.

For rigid models, the intramolecular energy is zero. For flexible models, separate gas phase simulations were performed for 5 ns with a single water molecule in a box of the same size as the corresponding liquid simulation. The gas phase simulation used the stochastic scaling thermostat^31^ with a 0.5 fs relaxation time as a standard Nosé–Hoover thermostat may have problems with such an isolated molecule.

The *D* was computed using the Einstein formula from a 5 ns NVT simulation using a 1728 water box at 298 K. The temperature was controlled with the Nosé–Hoover thermostat using a 5 ps relaxation time. The mean square displacement was fitted from 5 to 15 ps.

## IV. RESULTS AND DISCUSSIONS

Figure 2 shows the radial distribution functions for the three versions of each model. For the O–O RDFs, the EG and the corresponding flexible (FL) models are superimposable. The MG model has almost the same peak locations but the first O–O peak is slightly lower by 0.2. This is probably a result of the slightly reduced dipole moment for the MG model. A similar trend can be seen with the O–H RDF, although in this case, a minor difference can also be seen between the EG and FL models with the FL models having a slightly lower first peak. It is worth noting that this is not the intramolecular OH peak, but the intermolecular peak. The intramolecular vibration being captured by the FL model leads to a very small broadening of the first intermolecular peak associated with the hydrogen bond. Overall, it is probably safe to conclude that ignoring intramolecular vibrations has little effect on the intermolecular RDFs. The MG and EG models behaved similarly with the MG having a minor reduction of the height for the first peak.

Various properties of the water models are summarized in Table III. The *ΔH*_*vap*_ of the flexible models is in closer agreement with the EG models, which is about 3 kJ/mol higher than the corresponding MG model. The better agreement between the FL and EG models is actually an interesting cancellation of the larger polarization self-energy correction of the EG model with the intramolecular distortion energy of the FL model. It should be noted that none of the rigid models have intramolecular energies. The average intramolecular energy of the FL model is not the same in the liquid and the gas phases. While the FL model assumes the shape of the model in the gas phase, its average shape is that of the EG model in the liquid. The geometrical distortion causes the average intramolecular energy to be larger for the liquid when compared to the gas, which will be referred to as the intramolecular distortion energy. In a sense, this distortion energy is a polarization self-energy as the FL model is polarized by closing the HOH angle and stretching the OH bond in the condensed phase. Thus, it is not entirely surprising that the intramolecular distortion energy of the FL model is of a similar magnitude when compared to the increased polarization self-energy of the EG model.

The diffusion constants show a fairly large variation with respect to the FL, MG, and EG models. In each case, the EG model reduces the diffusion constants by 10%–15% and the diffusion constant of the MG model is a substantial 25%–65% higher when compared to the corresponding FL model. The faster diffusion for the MG models is easy to interpret as the models have a slightly smaller dipole moment, leading to a slightly weaker hydrogen bond, and hence faster hydrogen bond rearrangements. The smaller diffusion constant for the EG models when compared to the FL models probably reflects the role of flexibility in the diffusion process. OH vibration should transiently weaken a hydrogen bond and facilitate hydrogen bond rearrangements, which leads to a faster rate of diffusion for the FL models relative to the EG models.

For surface tension, *γ*, the difference between the EG models and the corresponding FL models is small, with the EG model providing a *γ* about 1 mN/m higher for the BLYPSP-4F and WAIL models and 2 mN/m higher for q-TIP4P/F. The reduction in *γ* for the MG model is more substantial, being 5% for the two AFM based models and 10% for q-TIP4P/F.

For *ε*, the difference is rather small between the FL and EG models for the two AFM based potentials. It is worth noting that the scaled *ε* of the EG models is slightly smaller than the corresponding FL model, leading the EG models to be in better agreement with experiments. We note that the small reduction in *ε* leading to a better agreement with experiments may not be completely fortuitous. The *ε* is proportional to the fluctuation in dipole moments. With the OH bond fluctuating between the classical turning points without proper treatment of quantum effects, it is possible a FL model would overestimate dipole fluctuations, thus slightly overestimate *ε*. However, a path integral calculation would be needed to verify such a hypothesis.

The q-TIP4P/F EG model has a substantially smaller *ε* when compared to the FL model. The *ε*_*o*_ for the FL version of the q-TIP4P/F model is in good agreement with that reported previously.^10^ The reason for the large difference in *ε* between FL and EG in case of q-TIP4P/F is not entirely clear to us. It has been pointed out previously that a repulsion term between the water hydrogen and the site carrying the negative charge is important for water models due to the breakdown of the point charge approximation at short distances.^42^ While both WAIL and BLYPSP-4F have such a term, the q-TIP4P/F model does not have repulsion between hydrogen and the M-site. It is possible that large dipole fluctuations are associated with very short hydrogen bonds formed with the q-TIP4P/F model without such repulsion, and the problem is exacerbated when the OH bond becomes flexible.

For all three water models, the corresponding MG models give a *ε* around 10% smaller than the corresponding EG models. This trend is consistent with the 4%–5% smaller dipole moments of the MG models when compared to EG.

*Cv* shows the largest difference between rigid and flexible models, while the difference between different force fields is smaller. This can be explained by the equipartition theorem. If the interactions are fully harmonic, the *Cv* is only determined by the degrees of freedom (DOF) available to the system. The difference between the various force fields is a result of anharmonicity, and the difference between the flexible and rigid versions of each force field is a result of losing the three intramolecular DOF in the rigid models. The equipartition theorem suggests the difference should be 3R, which is 25 J/K mol. The observed difference between the EG and FL models is very close to 25 J/K·mol, consistent with the expected value. The EG version of the two AFM based models gives *Cv* that is in very good agreement with experiments, which is not surprising considering the quantum nature of the intramolecular vibrations is better approximated with such rigid models.

The difference in *Cv* between the MG and EG models is very small for the two AFM based models, with the MG *Cv* slightly lower than that of the EG by 3–4 kJ/mol. The difference is 10 J/K·mol for q-TIP4P/F. The larger difference is probably a result of the fact that the q-TIP4P/F model has more anharmonicity in the intermolecular surface with the sharply repulsive 1/*r*^12^ term.

It is interesting that for almost all properties shown in Table III, the difference between the MG and the FL versions of the q-TIP4P/F model is larger than the corresponding differences between the MG and FL version of the AFM models. The difference in the intramolecular bond and angle potentials is quite small between the AFM and the q-TIP4P/F potentials. We thus attribute the larger difference to the use of the 1/*r*^12^ repulsion term and the lack of repulsion between hydrogen and the site carrying a negative charge in the q-TIP4P/F potential. The form of intermolecular energy used in q-TIP4P/F is quite popular among water potentials; we thus anticipate other potentials sharing similar intermolecular energy expressions to behave like q-TIP4P/F.

**FIG. 3.**
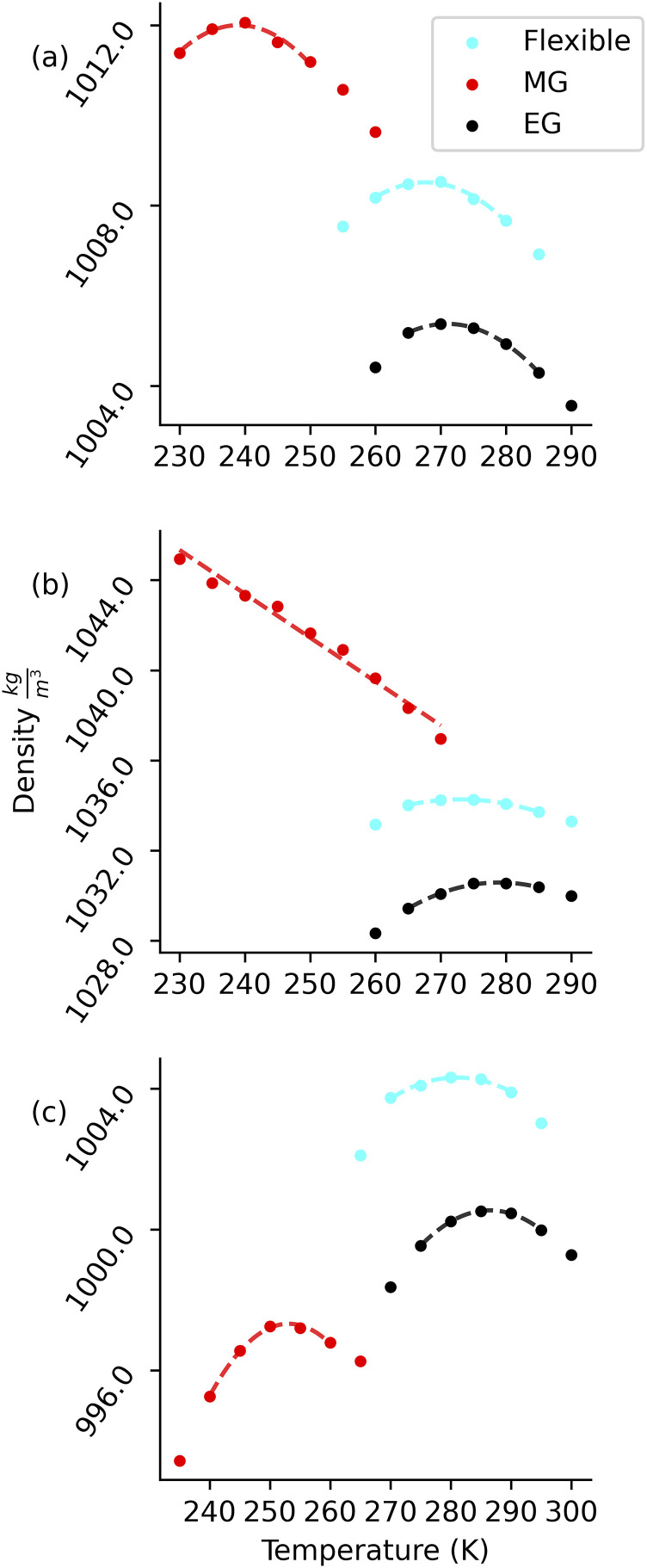
Density as a function of temperature for (a) BLYPSP-4F, (b) WAIL, and (c) q-TIP4P/F models. Except for the MG WAIL model, the dashed line is the region fitted to determine TMD. For MG WAIL, a TMD cannot be observed in this temperature range.

The temperature dependence of density for each model is shown in Fig. 3 with the temperature of maximum density (TMD) reported in Table III. The TMD of the EG models is 4–6 K higher than that of the corresponding FL models. It is interesting that the MG models with only slightly lower dipole moments have a density profile quite substantially different from the corresponding FL models. The TMD of the MG models is around 28 K lower than that of the corresponding FL model for both BLYPSP-4F and q-TIP4P/F.

**FIG. 4.**
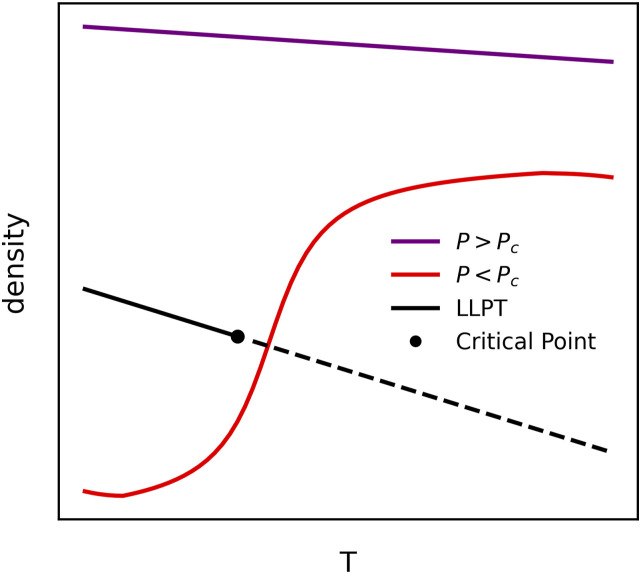
Schematic picture showing the density and temperature diagram at pressures above and below the putative liquid–liquid critical point. Since the putative liquid–liquid phase transition line has a negative slope with the Widom line below the critical point, a TMD is expected only at pressures below the critical pressure *P*_*C*_. If *P*_*C*_ is in the negative pressure, the TMD would disappear.

The TMD of the MG version of WAIL water cannot be found in the temperature range studied. The leading hypothesis for the existence of the TMD has been attributed to a second liquid–liquid critical point in supercooled water.^43–45^ With the second critical point hypothesis, the presence of the lower density form of water near the Widom line is responsible for the density anomaly. Previous simulations showed that the putative Liquid-Liquid Phase Transition (LLPT) line in supercooled water has a negative slope^46,47^ with a first order phase transition above the critical point and the Widom line below the critical point in the *T*–*P* diagram. While the TMD can be observed as water approaches the Widom line,^45^ no density anomaly is expected if water approaches a phase transition line at a pressure above the critical pressure, *P*_*C*_, as shown in Fig. 4. It is possible that with the reduced dipole moment, the *P*_*C*_ of the MG version of the WAIL model could become negative.^48^ In that case, a simulation at 1 bar pressure would not show a density anomaly as it is now above the critical point and away from the Widom line.

The *T*_*M*_ of the MG ice is 15–20 K lower than that of the corresponding FL ice for the three models. It is interesting that the *T*_*M*_ of EG ice is 3–5 K higher than that of the corresponding FL ice. While the 3–5 K difference is not much larger than the uncertainty, it is worth pointing out that the EG *T*_*M*_ is consistently higher than the FL *T*_*M*_ for all three models. This suggests that the trend of the EG model having a slightly higher *T*_*M*_ may not be accidental. The 5 K difference in the case of WAIL brings the WAIL *T*_*M*_ to even better agreement with experiments.

As mentioned previously, the classical Hooke’s picture of the OH vibration is not correct considering the quantum nature of this high frequency mode. The QM ground state has its probability maximum at the equilibrium bond length rather than the vibrational turning point as in the classical Hooke’s picture. The EG model thus better approximates the most probable QM geometry when compared to the FL model. We argue that the slightly improved agreement of the *T*_*M*_ for the EG WAIL model may not be accidental. On the other hand, with a lack of understanding of the mechanism for the higher *T*_*M*_, there is no proof that proper modeling of quantum nuclear effects will cause the WAIL model to produce an improved *T*_*M*_. Performing path integral calculations to determine the *T*_*M*_ is very challenging. Presumably, the effective centroid potential^49^ can be used to obtain a converged estimate. However, even such a simulation would still be quite challenging and is beyond the scope of this work.

## V. SUMMARY AND CONCLUSION

A flexible water model requires the use of small time steps. There are cases where it is desirable to simulate a rigid version of a flexible model to improve computational efficiency. This work shows that simply constraining the geometry of the rigid model using the bond length and bond angle of the flexible model will lead to a model (the MG model) that behaves quite differently from the flexible model (the FL model). Several properties see quite substantial changes. For example, the diffusion constants of the MG models are 50% higher and the TMD of MG models shifts down by 30 K and becomes undetectable in the case of WAIL. In the majority of the cases, the cause for the different performance is not due to rigidity but the difference in the mean dipole moments in water. This is supported by the EG models showing much closer agreement with the FL models for most properties, except for the diffusion constant and heat capacity.

For the two AFM models, where the model was developed solely by fitting to high quality electronic structure calculations, most properties of the corresponding EG models are better than those of the FL model. Properties that show an improvement include heat capacity *Cv*, surface tension γ, dielectric constant *ε*, and melting temperature *T*_*M*_. While for *Cv*, the improvement of 3R is obviously the result of the EG models’ ability to better capture the QM nature of the highly quantized OH vibrations, some of the improvements in other properties could be fortuitous. However, these could also be perceived as evidence that proper treatment of nuclear quantum effects could be important for the proper modeling of these properties.

In addition, while we attribute the difference between the MG and EG models to a change in the dipole, other factors may also play a role. For example, the small geometry change could affect the hydrogen bond length, and the small angle change could affect tetrahedrality. Careful delineation is not easy and will be worthy of careful study in the future.

We note that various efforts have been made to create flexible versions of rigid models. In most cases, the equilibrium bond length and angles for such flexible models were taken from the rigid models. In such a case, the mean dipole of the flexible water model created will be higher than that of the corresponding rigid model in condensed phase simulations, similar to the relationship between the MG and FL models in this study. Thus, care has to be taken when creating flexible versions of rigid models. The parameters of the flexible models should be adjusted to mimic the relationship between EG and FL models to ensure the flexible models behave similarly when compared to the rigid ones.

The simulation showed an interesting disappearance of TMD for the MG version of the WAIL potential, which could be attributed to the putative liquid–liquid critical point of this model residing in the negative pressure regime.

In addition, a new NVE three phase coexistence method is presented in this work, which is able to compute the *T*_*M*_ of the water models without the use of any thermostats and barostats. This method eliminates potential artifacts from the thermostats and barostats and demonstrates good stability in determining *T*_*M*_.

## SUPPLEMENTARY MATERIAL

The intermolecular component of the configuration energy, the self-polarization energy, parameters for the three models simulated, and a graph showing the total energy as a function of time during the NVE three-phase coexistence simulation can be found in the supplementary material. Six ice boxes along with the corresponding LIL slabs are also provided in the supplementary material.

## Data Availability

The data that support the findings of this study are available from the corresponding author upon reasonable request.
